# Women’s perspectives on ultrasound as primary imaging modality for focal breast complaints: a qualitative study

**DOI:** 10.1186/s13244-025-01928-4

**Published:** 2025-02-26

**Authors:** Carmen C. N. Siebers, Linda Appelman, Mette Palm, Linda Rainey, Mireille J. M. Broeders, Ritse M. Mann

**Affiliations:** 1 Department of Medical Imaging, Radboud University Medical Center, Nijmegen, The Netherlands; 2 Department for Health Evidence, Radboud University Medical Center, Nijmegen, The Netherlands; 3 Dutch Expert Centre for Screening, Nijmegen, The Netherlands; 4 Department of Radiology, The Netherlands Cancer Institute, Amsterdam, The Netherlands

**Keywords:** Breast, Cancer, Digital breast tomosynthesis, Breast ultrasound, Patient perceptions

## Abstract

**Objectives:**

The breast ultrasound trial (BUST) demonstrates a high negative predictive value of ultrasound for women presenting with focal breast complaints, suggesting a potential shift from mammography/digital breast tomosynthesis (DBT) to ultrasound as a primary imaging modality. This BUST side-study explored women’s perspectives on adopting ultrasound as the primary diagnostic tool.

**Methods:**

Twenty-nine female BUST participants (mean age = 48.4, SD = 8.3) with benign findings after diagnostic evaluation participated in one of six focus group interviews 18–24 months post-imaging. Discussions were transcribed and analyzed thematically.

**Results:**

Four overarching themes were identified; personal health situation, organization of breast care, effectiveness of imaging, and professionals’ attitudes and roles. Participants considered their own health history and complaint type (personal health situation) and discussed eligibility for national screening programs and the costs of both exams (organization of breast care). Opinions varied on the effectiveness of imaging, particularly regarding the importance of detecting additional non-symptomatic findings with mammography/DBT that may be missed by ultrasound. Concerns were also raised about implementing research findings without conclusive scientific evidence. Health professionals’ attitudes and roles encompassed the influence of GPs’ and radiologists’ attitudes and the process of image interpretation.

**Conclusion:**

These findings reveal diverse attitudes of women towards ultrasound as a primary modality, warranting caution when transitioning to new clinical standards. Providing comprehensive information about the evidence on the benefits and risks of different imaging modalities is essential, and fostering shared decision-making could enhance acceptance. Offering women the choice of additional imaging, such as mammography/DBT after an initial ultrasound, may balance clinical performance with patient autonomy.

**Critical relevance statement:**

Patients’ perspectives on medical procedures are increasingly significant in modern healthcare. Women’s perceived barriers and facilitators to diagnostic imaging, shaped by numerous factors, offer healthcare professionals insights beyond pure biomedical approaches, fostering shared decision-making within radiology and other clinical contexts.

**Key Points:**

- This study explores women’s perspectives on breast ultrasound as a primary imaging modality.
- Attitudes towards an ultrasound-first approach are shaped by internal and external considerations.
- Considerations are highly informed by emotional responses and lack of knowledge.
- Insights into women’s perspectives inform healthcare professionals and foster shared decision-making within radiology.

**Graphical Abstract:**

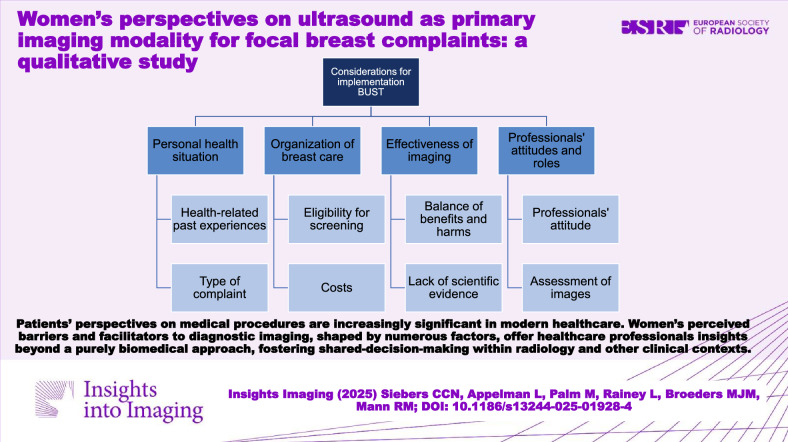

## Introduction

Every year, tens of thousands of women visit a hospital’s radiology department with breast complaints, such as lumps, focal pain, or nipple and skin abnormalities. In the Netherlands, for women > 30-years-old mammography (mostly digital breast tomosynthesis (DBT)) is the standard first-line imaging modality for breast evaluation, followed by targeted ultrasound (US) [1]. However, in the past decades, multiple studies have been done to investigate the potential of targeted US as a primary diagnostic test, showing high sensitivity and specificity with US as a stand-alone modality [2–5]. However, these studies were mainly focused on the younger female population (under 40 years or 45 years).

The prospective breast ultrasound trial (BUST) was designed to investigate the diagnostic accuracy of US in women of all ages. This study showed high diagnostic accuracy of US in symptomatic women (sensitivity of 98.5% and specificity of 90.7%), with only limited added value of additional DBT (0.2%) for the evaluation of the focal complaints. In 0.4% of the women malignant incidental findings in asymptomatic areas were detected [6]. Therefore, we interpret these results as promising evidence for US as the first-line diagnostic test in terms of clinical performance. In fact, the European Society of Breast Imaging has recently recommended the initial US in women between 30-years-old and 40-years-old [7]. However, little is known about women’s perspectives on the implementation of this alternative clinical pathway.

Several studies have previously been done on women’s perceptions of US and DBT and US-only diagnostics in the clinical setting. Although these studies give some insight into women’s attitudes towards US-based diagnostics, the information provided is still limited. First, the data were largely quantitative, preventing women from fully expressing their thoughts and ideas. For example, we found that the majority of women’s motivation to visit the breast clinic was to get their complaint explained (88.3%), vs 3.2% who wished to “check” both breasts. Also, most women (82.4%) reported they would be sufficiently reassured when the initial US showed benign findings [8]. However, it remains unclear which considerations are involved for women to reach these conclusions. Moreover, the parts of the surveys that did provide qualitative data only focused on women’s experiences and perceptions of US and DBT, without diving deeper into their perspective on the implementation of US as a first-line imaging modality in symptomatic women [9].

Therefore, in this side-study to the BUST we organized focus group (FG) interviews with women who previously participated in the BUST, aiming to explore their barriers and facilitators towards the implementation of US as a primary imaging modality in the clinical setting.

## Methods

### Design

This study adopted an FG methodology to examine women’s attitudes toward US as the primary diagnostic test for breast imaging. To gather this information, a semi-structured interview guide was developed. Ethical approval was waived by the ethical committee CMO Arnhem-Nijmegen (2016-3034). All participants provided written informed consent for both the BUST study and FG participation.

### Subjects

FG participants were former BUST study participants who visited Radboudumc Nijmegen or St. Antonius Hospital Utrecht between September 2018 and March 2019 and had undergone US first, followed by DBT, for breast evaluation. All participants consented to be approached for follow-up studies. Recruitment for the FGs took place 18–24 months post-imaging, involving phone calls and emails. Women of different ages (all > 30 years) and with various BI-RADS scores (BI-RADS 1/2 or BI-RADS 3 with negative biopsy or follow-up results) were included to capture diverse perspectives. Only those with benign findings after imaging were invited to participate in FG discussions, as women diagnosed with breast cancer would have proceeded directly to DBT, making US-only diagnostics irrelevant for them.

### Procedure

Participants signed an online informed consent and completed a short questionnaire. FG interviews were held via Zoom Meetings, facilitated by the first author, with a maximum of six participants per session, lasting 90–120 min. Each session included a background presentation on the BUST, a break, and a summary of BUST results to further guide discussions.

### Statistical analysis

FG discussions were transcribed verbatim and analyzed in Atlas.ti, using thematic analysis following Braun and Clarke’s steps: data familiarization, coding, theme development, reviewing, defining and naming, and final analysis [10]. Two researchers (C.C.N.S. and M.P.) independently coded and reviewed the data, resolving any discrepancies through discussion. Descriptive statistics were analyzed using IBM SPSS Statistics 25.

## Results

### FG participants

Six FGs were conducted between October and December 2020, involving 29 women aged 33 years to 63 years (M = 48.4, SD = 8.3). Each FG included three to six participants, with one woman leaving during the session. Participants had diverse marital statuses, educational backgrounds, and origins. Many had prior experience with the national screening program or planned future participation, and nearly all had encountered breast cancer within their personal circles. Participant characteristics are summarized in Table 1.

**Table 1:** Participants’ characteristics

	*N*	(%)		*N*	(%)
Age category			Origin		
30–39	4	(13.8)	Dutch	23	(79.3)
40–49	12	(41.4)	Turkish	1	(3.4)
50–75	13	(44.8)	Caucasian	1	(3.4)
Hospital			Spanish	1	(3.4)
Radboudumc	16	(55.2)	Moroccan	1	(3.4)
St Antonius hospital	13	(44.8)	Italian	1	(3.4)
Marital status			Missing	1	
Married	19	(65.5)	Past screening participation		
Single	4	(13.8)	Yes	16	(55.2%)
Living together	3	(10.3)	No	12	(41.4)
Divorced	1	(3.4)	Missing	1	
Widowed	1	(3.4)	Future screening participation		
Missing	1		No	25	(86.2)
Education			Yes	3	(10.3)
Secondary	3	(10.3)	Missing	1	
Vocational	5	(17.2)	Breast cancer in a personal environment		
Higher vocational	11	(37.9)	Yes	24	(82.8)
University	7	(24.1)	No	4	(13.8)
Missing	3		Missing	1	

### Description of themes

**Fig. 1:**
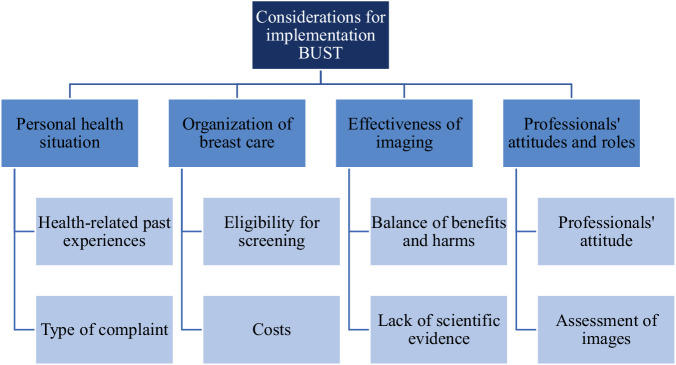
Overview of the (sub) themes associated with women’s considerations for implementation of initial targeted US

Four overarching themes emerged from the data: Personal health situation, Organization of breast care, Effectiveness of imaging, and Professionals’ attitudes and roles (Fig. 1).

#### Personal health situation

Health-related past experiences: Women’s opinions on the US as the primary diagnostic test were influenced by past experiences with imaging or health issues, including unclear US findings, discrepancies between US and DBT results, detection of (benign) additional lesions with DBT, prior medical errors, breast implants, or a personal/family cancer history. These experiences often led to a preference for DBT:

“In my case, that one claim went wrong in the past, so to say, the exact opposite was shown. So purely following that experience, I think I have no faith and would want to go through the whole examination”. (ID7, FG2, age 33)

Conversely, unpleasant DBT experiences, previous breast reduction or biopsy (but DBT was redundant after biopsy), dense tissue, or lesions invisible at DBT fostered a preference for US:

“I have the experience that after mammography something unclear was seen, and during ultrasound it became clear that it was nothing. […] I have more faith in ultrasound because of that”. (ID11, FG2, age 61)

Type of complaint: Women highlighted their complaint type as crucial. For instance, they believe that frequent cysts visible in US may not require DBT, but suspicious or first-apparent complaints might. Additionally, although currently the protocol prescribes DBT and targeted US for evaluation of complaints, and deviating from this protocol is only induced by the radiologist, women strongly emphasize the importance of personal choice:

“That you indeed would have the choice of… gosh I would still rather have mammography as well, because otherwise, I still don’t have faith in it. That would be pleasant”. (ID14, FG3, age 52)

#### Organization of breast care

Eligibility for screening: Women considered their eligibility for the national breast cancer screening program as important, with some feeling that biennial screening reduced the need for DBT in the diagnostic setting:

“If you are over the age of 50 you get regularly screened anyway”. (ID14, FG3, age 52)

However, others stressed the limitations of the program, including gaps in coverage for younger women who are also at risk, lack of assured participation, rapid tumor progression, and the inability of screening methods to detect all cancers:

“You know, it is only a snapshot, […] because one month later it can be totally wrong”. (ID25, FG5, age 37)

Additionally, women expressed a strong desire for additional information on screening program reliability, radiologist access to screening results, and the possibility of lowering the screening age, as these factors would affect their desire for DBT in the diagnostic setting.

Costs: From a financial perspective, women felt US-only diagnostics could reduce costs and improve accessibility:

“I can imagine that for less fortunate people it could be a barrier of some sort. If you would only have to do an ultrasound, than it is cheaper of course”. (ID29, FG6, age 41)

#### Effectiveness of imaging

Balance of benefits and harms: The identification of additional findings through DBT, which would remain undetected with US, was a common concern. The majority perceived the number of patients with additional detected malignant lesions in BUST as quite extensive, praising DBT for its ability to detect these malignancies, and expressing empathy for those in whom such lesions might have been missed:

“What if it were you, what if it were your daughter, you know…”. (ID26, FG6, age 35)

Others, however, viewed DBT as overdiagnosis in this context:

“In that case, the only reason for doing mammography would be as an early population screening. […] If that is the reason to let women undergo this as standard, than I would say, no why would you…”. (ID22, FG4, age 44)

The rate of additional false-positive findings with DBT was also debated. While some women suggested that these false positives only cause anxiety of having breast cancer, others would accept them to avoid missed cancers:

“It’s better to have a lot of stress for a week and to hear that it’s OK, than having no stress and after a certain period of time cancer reveals itself”. (ID4, FG1, age 46)

Additionally, women felt a great need for additional information on e.g., motives for conducting BUST, and the strengths, limitations, and clinical performance of US and DBT. Some confused skipping diagnostic DBT with omitting mammography from screening. Others were unsure if benign lesions could become malignant or if DBT could harm the breasts. Additionally, women inquired about whole-breast US or MRI as alternatives to DBT, suggesting these modalities replace DBT if possible. Some also recommended limiting DBT to high-risk groups or improving its comfort.

Lack of scientific evidence: Participants emphasized the need for scientific validation of US as a standalone modality:

“I think it is important that it is scientifically substantiated, like with this study”. (ID14, FG3, age 52)

For now, some women remain hesitant about US, as it is still in the research phase, and DBT is typically the standard:

“Now, when you have a lump, you expect, hop mammography. That’s what we, as women, think is good”. (ID15, FG3, age 47)

#### Professionals’ attitudes and roles

Professionals’ attitude: Women highlighted the role of GP and radiologist confidence in US diagnostics. For example, a hesitant GP might lead to a preference for additional DBT:

“In my case, the GP said, well, I think it’s nothing, but we won’t take any risk. Keeping that in mind, I don’t think it’s unpleasant at all to only get an ultrasound. […] But if the GP doubts, then I don’t know whether I would be just as reassured if there is only an ultrasound”. (ID29, FG6, age 41)

Regarding radiologists, women valued clear communication and physician confidence:

“They can explain very well why an ultrasound would be or would not be sufficient. And as a patient, if you give me that information, I don’t necessarily need a mammography after that, I think”. (ID22, FG4, age 44)

Assessment of images: There was a general lack of women’s knowledge on whether US images are captured and stored for later review, and who assesses the images, with confusion regarding the radiologist being a physician or a radiographer. While most trusted radiologists’ skills, some questioned whether assessment could vary based on the radiologist’s experience or mood, suggesting that two radiologists review the images:

“I’m going to a specialist who will take a good look at me. I have faith in that”. (ID9, FG2, age 55)

“Maybe I would find it very reassuring if two experts looked at it to keep each other alert”. (ID15, FG3, age 47)

## Discussion

This study explored women’s perceptions of using US as a primary imaging modality in the clinical setting. The findings reveal considerable variation in acceptability, with women considering factors such as their personal health situation (e.g., health history and complaint type) and the organization of breast care, including eligibility for screening and exam costs. The effectiveness of imaging, including DBT’s ability to detect incidental findings and the scientific evidence supporting standalone US, was considered crucial. Additionally, professionals’ attitudes, particularly those of GPs and radiologists, and the image assessment process were important. Furthermore, women’s information needs were emphasized, as knowledge about these factors influenced their preference for DBT in the diagnostic setting.

Women’s personal health situation and the type of complaint they presented with appeared to influence their perceived need for either a whole-breast evaluation or a complaint-focused approach. This likely reflects the extent to which women feel at risk for breast cancer, with those perceiving a higher risk preferring more extensive examinations when presenting with complaints, compared to those who feel their risk is low [11].

A notable factor in women’s perceptions of an US-only approach was eligibility for the national breast cancer screening program. While the Dutch program focuses on asymptomatic women aged 50–75, and diagnostic imaging is used for evaluating symptomatic complaints, some women linked the two types of exams. They viewed DBT in the diagnostic setting as largely redundant given biennial mammograms in the screening program, but were reluctant to omit diagnostic DBT before reaching screening eligibility. This suggests that women see diagnostic DBT as an opportunity for early breast control. Some also expressed dissatisfaction with the 50-year age cutoff for screening, citing the incidence of breast cancer in younger women. This aligns with other studies showing that women from the age of 40 tend to undergo early mammography before the recommended age of 50, due to a lack of confidence in the guidelines [12].

Women’s preferences for an US-first approach were primarily based on the effectiveness of the imaging. They weighed the benefits and harms of the exams, emphasizing the need for extensive scientific evidence on US performance before implementing this approach, as DBT is currently seen as the gold standard. Some participants suggested replacing DBT with MRI or whole-breast US, reflecting concerns about missing breast cancer in other areas when presenting with a focal complaint. This aligns with literature showing that women fear underdiagnosis more than overdiagnosis in the diagnostic setting [13, 14]. Skepticism about omitting DBT stemmed from concerns about missing asymptomatic tumors, highlighting that women prioritize outcome features (quality of the test) in addition to process features (experience of the test) [13]. This concern likely reflects a fear of breast cancer rather than discomfort with the procedure itself, despite DBT being considered uncomfortable by many women [15].

Medical professionals played a crucial role in women’s satisfaction with an US-first approach. Participants noted that the hesitancy of GPs regarding the presence of breast cancer and the confidence of radiologists in US performance influenced their sense of safety with the performance of US only. This is consistent with the literature, showing that a positive professional attitude can reduce patient anxiety and affect the perception of the severity of complaints [16, 17]. These findings underscore the importance for radiologists to be mindful of the impact their approach can have on patients.

Women expressed significant information needs regarding the clinical performance of US and DBT, image assessment procedures, the roles of radiologists and radiographers, and broader breast-related topics (e.g., the potential for benign lesions to become malignant). These findings are supported by studies showing that women, particularly in the younger population (< 44–50 years), often lack detailed knowledge about mammography and the roles of radiology professionals [18–23]. Providing women with detailed information—such as through leaflets distributed by GPs or educational videos in hospital waiting rooms—has been shown to improve their breast-related knowledge and understanding of radiology professions, and reduce pre-exam anxiety [24, 25]. These interventions could help address gaps in knowledge about diagnostic procedures.

In an era marked by constrained healthcare resources and the growing emphasis on personalized screening, this approach has the potential to significantly reduce costs in symptomatic settings, thus enabling the reallocation of resources for broader implementation in screening programs. However, should the current standard of routine DBT for all eligible individuals shift toward a more selective, risk-based approach, clear communication of its benefits, such as improved diagnostic precision and optimized resource allocation, will be essential. Furthermore, maintaining public trust in this new methodology will be crucial, as the majority of patients currently regard DBT as the standard of care.

Indeed, our study reveals significant variation in women’s preferences for diagnostic breast US alone or with additional DBT. While some women trusted the radiologist’s expertise in selecting the most appropriate diagnostic test, the need for autonomy in decision-making was emphasized. Although shared decision-making (SDM) is common in treatment choices, it is less frequently applied to diagnostic test options. This is crucial, as SDM is increasingly relevant in radiology [26]. However, women need basic knowledge of risks and the ability to understand the consequences of their decisions, determining their ability and ‘readiness’ to be involved in SDM. Additionally, SDM may be more challenging for older women, those with lower education or non-western backgrounds, or when the clinician-patient relationship is underdeveloped [27]. However, these challenges should not exclude women from SDM but highlight the need for better support and preparation, such as providing accessible information and fostering a strong doctor-patient relationship [26]. While patient decision-making can be complex when many options are involved [27], our study’s context offers only two choices, facilitating SDM. We, therefore, believe that women should be able to consider and be supported in making an informed decision between the US alone or additional DBT for the evaluation of breast complaints.

The present study is the first to explore women’s perceptions regarding an US-only diagnostic pathway. However, caution is needed in interpreting our findings due to potential selection bias, as participants had prior experience with diagnostic imaging in the BUST study. All participants were familiar with both US and DBT, which may differ from the perceptions of first-time attendees. Additionally, since all women underwent DBT, their views on US-only diagnostics may have been influenced by the absence of clinical consequences as both modalities were used, and by the radiologists’ beliefs and communication. Our sample was also quite modest and limited to women in the Netherlands, where current guidelines recommend diagnostic DBT for women over 30 years and breast screening for those aged 50–75. Thus, our findings are specific to women familiar with these guidelines, and may differ from populations in countries with different age cut-offs. Future studies involving larger, unbiased patient samples from diverse international settings would provide valuable comparative insights.

## Conclusion

Our study reveals significant variation in women’s preferences for diagnostic breast imaging using US and DBT. Women value not only the process but also the quality of the diagnostic tests. Since perceptions are influenced by emotional responses and lack of knowledge, providing educational material on breast symptoms and imaging procedures is essential. For instance, GPs could provide information leaflets after consultations, or educational videos could be shown in hospital waiting rooms. While enhancing women’s knowledge is important, it should complement, not replace, the radiologist’s expertise. This can support SDM in breast radiology, which should include discussions of the benefits and drawbacks associated with US and DBT, as women are willing to weigh these trade-offs.

## Data Availability

The raw data for this study consists of FG interview transcripts, which may contain sensitive participant information. Participants did not give explicit consent for the data to be shared in the public domain, therefore we are unable to publicly share it. Relevant de-identified excerpts of the transcripts are provided in the paper. Full data are available from the METC Oost-Nederland (WMO) (contact via METCoost-en-CMO@radboudumc.nl) for researchers who meet the criteria for access to confidential data.

## Abbreviations

BUST: Breast ultrasound trial
DBT: Digital breast tomosynthesis
SDM: Shared decision-making
US: Ultrasound
